# Retinal Image Enhancement Using Cycle-Constraint Adversarial Network

**DOI:** 10.3389/fmed.2021.793726

**Published:** 2022-01-12

**Authors:** Cheng Wan, Xueting Zhou, Qijing You, Jing Sun, Jianxin Shen, Shaojun Zhu, Qin Jiang, Weihua Yang

**Affiliations:** ^1^College of Electronic Information Engineering, Nanjing University of Aeronautics and Astronautics, Nanjing, China; ^2^College of Mechanical and Electrical Engineering, Nanjing University of Aeronautics and Astronautics, Nanjing, China; ^3^School of Information Engineering, Huzhou University, Huzhou, China; ^4^The Laboratory of Artificial Intelligence and Bigdata in Ophthalmology, The Affiliated Eye Hospital of Nanjing Medical University, Nanjing, China

**Keywords:** image enhancement, convolutional neural network, retinal image, deep learning, generative adversarial network

## Abstract

Retinal images are the most intuitive medical images for the diagnosis of fundus diseases. Low-quality retinal images cause difficulties in computer-aided diagnosis systems and the clinical diagnosis of ophthalmologists. The high quality of retinal images is an important basis of precision medicine in ophthalmology. In this study, we propose a retinal image enhancement method based on deep learning to enhance multiple low-quality retinal images. A generative adversarial network is employed to build a symmetrical network, and a convolutional block attention module is introduced to improve the feature extraction capability. The retinal images in our dataset are sorted into two sets according to their quality: low and high quality. Generators and discriminators alternately learn the features of low/high-quality retinal images without the need for paired images. We analyze the proposed method both qualitatively and quantitatively on public datasets and a private dataset. The study results demonstrate that the proposed method is superior to other advanced algorithms, especially in enhancing color-distorted retinal images. It also performs well in the task of retinal vessel segmentation. The proposed network effectively enhances low-quality retinal images, aiding ophthalmologists and enabling computer-aided diagnosis in pathological analysis. Our method enhances multiple types of low-quality retinal images using a deep learning network.

## 1. Introduction

**Figure 1:**
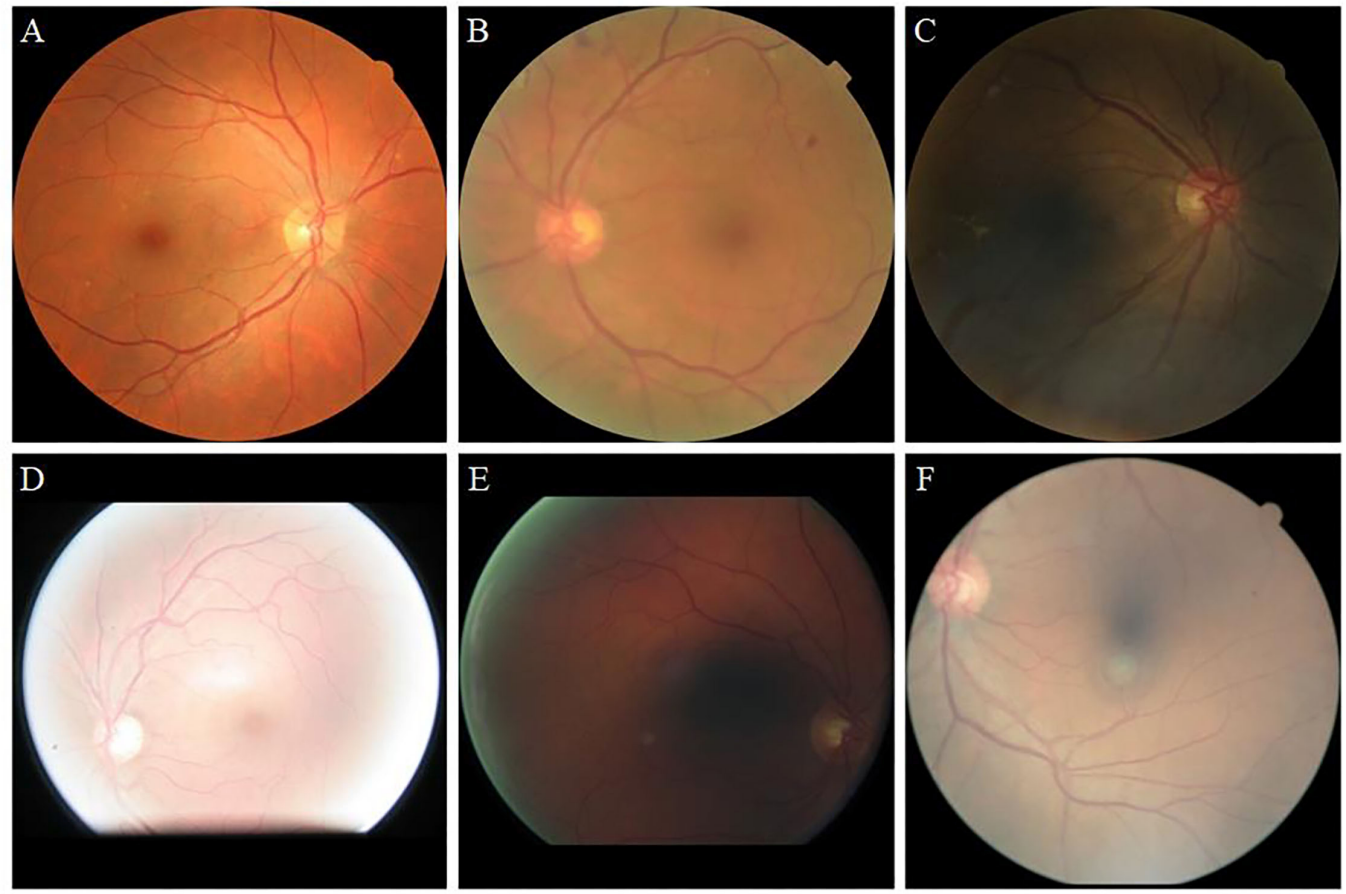
Retinal image instances. **(A)**: High-quality image. **(B)**: Blur. **(C)**: Low illumination. **(D)**: High illumination. **(E)**: Uneven illumination. **(F)**: Color distortion.

Retinal images are widely used for the screening and diagnosis of diseases, including diabetic retinopathy (DR) (1, 2), glaucoma (3, 4), and age-related macular degeneration (5, 6). These diseases often cause abnormalities in the blood vessels, optic cup, and optic disc. Only high-quality retinal images are capable of clearly showing the tiny blood vessels and optic disc profile. Low-quality retinal images, such as those shown in Figure 1, include blur, low illumination, high illumination, uneven illumination, and color distortion, and they limit the diagnostic capabilities of ophthalmologists and computer-aided diagnosis (CAD) systems (7). The enhancement of retinal images can clearly display fundus information including, blood vessels and optic disc, and increase the accuracy of CAD systems, such as the retinal vessel segmentation network.

Retinal image enhancement methods have been proposed by numerous researchers, and they can be categorized into histogram-based, fusion-based, and Retinex theory-based methods. Among the histogram-based methods, contrast limited adaptive histogram equalization (CLAHE) is a widely used method to enhance retinal images. The RGB channel, green model, and exponential histogram are the optimal choices when enhancing with CLAHE (8). (9) segmented the fuzzy histogram of the green plane and improved the average brightness of the image by using the intensity level of the equalizer sub-histogram. (10) obtained the brightness gain matrix by gamma correction and subsequently enhanced the contrast on the brightness channel with CLAHE. Setiawan (11) used histogram equalization to enhance the retinal image by increasing the difference between the maximum and minimum pixels. These methods equalize the gray-scale distribution of images and give rise to unnatural color transitions when processing retinal images. The fusion-based method integrates the image features under different conditions to enhance the original image. (12) used the transform domain algorithm to extract the background information image and fused it with the original retinal image. (13) separately extracted background brightness and foreground pixels of different intensities to effectively enhance blurred retinal images. These methods improve sharpness and can only enhance blurred retinal images. (14) used self-similar filtering to remove image noise. (15) generated different degrees of exposure images and used a weight matrix to fuse the original and the exposure image to obtain an enhanced result. The low-light image enhancement (LIME) method (16) combines the original brightness with prior knowledge to enhance the underexposed image from the perspective of increasing brightness. These networks are computationally intensive and slow in the processing of images. The Retinex theory (17) states that an image is the product of reflectivity and illumination. (18) removed the low-frequency illumination component of the retinal image and used single-scale retinex and multiscale retinex to enhance the retinal image before applying the classification of retinal vessels. The RETIC method (19) effectively enhances the low-contrast retinal image and further enhances the retinal blood vessels based on the hemoglobin image. However, the image enhanced by the RETIC method will generate additional noise, which must be further identified and removed (20). According to the Retinex theory, (21) performed gamma correction for the retinal image light-map and then combined it with CLAHE to make color adjustments in the brightness channel. (22) proposed the MSRCP method, which applied the MSRCR algorithm to the luminance channel to ensure that the pixel value of the enhanced image was between 0 and 255.

In recent years, learning-based methods have been widely applied in medical image processing, such as for the tasks of image classification, image segmentation, object detection, and localization (23). (24) used illumination regularization and structural loss to overcome the problem of uneven illumination in medical images, and enhanced endoscopic images, corneal confocal microscopy images and fundus images (25). The method they proposed was effective for low-illumination and high-illumination images, but did not apply to other low-quality fundus images, such as blur and color distortion. A method combining support vector machine and mathematical morphology was proposed to obtain a satisfactory classification accuracy in the quality filtered retinal image dataset (26). (27) combined vascular extraction with arteriovenous identification to achieve arteriovenous segmentation in retinal images using the U-Net semantic segmentation structure. (28) used the support vector machine to detect the optic disc for further diagnosis of glaucoma. These studies on retinal image processing are suitable for clear, high contrast images that can be processed automatically, and they perform better on high quality retinal image datasets. (29) used a data-driven method to enhance blurry retinal images. However, in general datasets, there are other types of causes for low-quality images, besides blur, that reduce the feasibility of processing retinal images. In our previous study (30), low-quality images with artificial noise were enhanced without distinguishing the categories of quality. Synthetic noise is easily learned for the convolutional neural network, which is essentially different from the low-quality images taken by the fundus camera.

In this article, we propose a learning-based method to enhance low-quality retinal images from datasets and clinics. The generative adversarial network (GAN) proposed by (31) realized the use of neural networks to generate pictures. However, GAN can neither be controlled by the user nor can it generate specific pictures. Based on GAN, the cycle-constraint adversarial network (CycleGAN) (32) used the cycle consistency to successfully separate the style and content of the image, hence maintaining the content of the image while changing its style. The enhancement of the retinal image from low to high quality is also image translation. We introduce convolutional block attention modules (CBAM) (33) into CycleGAN and propose a novel retinal image enhancement network Cycle-CBAM to enhance five types of low-quality images: blur, low illumination, high illumination, uneven illumination, and color distortion. Cycle-CBAM aims to eliminate the factors that lead to low quality and restore the original condition of retinal images.

The main contributions of this article study can be summarized as follows: (a) The ability of image style conversion prompted us to consider using CycleGAN for retinal image enhancement. (b) CBAM enhances the feature extraction ability of the network and intensifies the detailed information of the enhanced image. (c) Cycle-CBAM does not require paired images to train the network, which greatly reduces the difficulty of collecting images. (d) Our method uses the powerful feature extraction capability of deep learning to realize difficult image enhancement tasks.

## 2. Materials and Methods

In this section, we describe the methods employed to enhance the quality of retinal images. First, CycleGAN is applied for the style translation of retinal images to resolve the lack of paired low/high-quality images. Second, CBAM is introduced to solve the degeneration of texture and detail caused by training unpaired images. Third, to verify the enhancement effect of our method, the enhanced retinal image was applied to the retinal vessel segmentation network. Finally, the loss function and evaluation indicators used in our experiments are described.

### 2.1. Dataset

To train the retinal image enhancement network proposed in this study, we use the EyePACS (34) dataset and a proprietary dataset.

#### 2.1.1. EyePACS Dataset

The training set of the EyePACS dataset includes 35,126 color retinal images, of which 8,575 are low-quality and 26,551 are high-quality (34). The image resolution ranges from 433 × 289 to 5184 × 3456. We randomly select 500 low-quality and 500 high-quality images to construct the dataset for the image enhancement network. The selected images undergo a double-blind review of the image quality evaluation by three retinal ophthalmologists. The training set consists of 400 low-quality and 400 high-quality images. The remaining 100 low-quality and 100 high-quality images form the test set.

#### 2.1.2. Proprietary Dataset

The proprietary dataset is provided by the Affiliated Eye Hospital of Nanjing Medical University, including 17 sets of low/high quality paired color retinal images. These are preoperative and postoperative retinal images of cataract patients taken from the same perspective. Images were desensitized (anonymized) before being used in this study. Because different retinal image cameras were used, there are the following three image resolutions: 2736 × 1824, 1280 × 960, and 3456 × 2304. Since the photographs anonymization was applied before the study, informed consent from the patients was waived. Ethical approval has been obtained for the use of a proprietary dataset.

#### 2.1.3. Retinal Vessel Segmentation Dataset

The training set of the retinal vessel segmentation network consists of three public datasets: DRIVE (35), STARE (36), and CHASEDB1 (37). All these three datasets contain multiple color retinal images and their corresponding retinal vessel segmentation images. The DRIVE dataset contains 40 pairs of images with a resolution of 565 × 584, 30 pairs for training and 10 pairs for validation. The STARE dataset contains 20 pairs of images with a resolution of 700 × 605, 15 pairs for training and 5 pairs for validation. The CHASEDB1 dataset contains 28 pairs of images with a resolution of 999 × 960, 21 pairs for training and 7 pairs for validation. The 100 low-quality retinal images and their corresponding enhanced images with the CLAHE, fusion-based, MSRCP, LIME, CycleGAN, and Cycle-CBAM methods were used as the test set.

The images used in the image enhancement and retinal vessel segmentation network are all normalized and preprocessed to 512 × 512 resolution. The detailed information of the dataset in our study is shown in Table 1.

**Table 1:** Dataset details.

**Network**	**Dataset**	**Image Type**	**Number**	**Resolution**
Image Enhancement Network	EyePACS dataset	High/low quality color retinal images	35126 images	433 × 289 to 5184 × 3456
	Proprietary dataset	Preoperative and postoperative color retinal images of cataract patients	17 pairs images	2736 × 1824, 1280 × 960, 3456 × 2304
Retinal vessel segmentation network	DRIVE	Color retinal images and corresponding retinal vessel segmentation images	40 pairs images	565 × 584
	STARE		20 pairs images	700 × 605
	CHASEDB1		28 pairs images	999 × 960

### 2.2. Proposed Method

**Figure 2:**
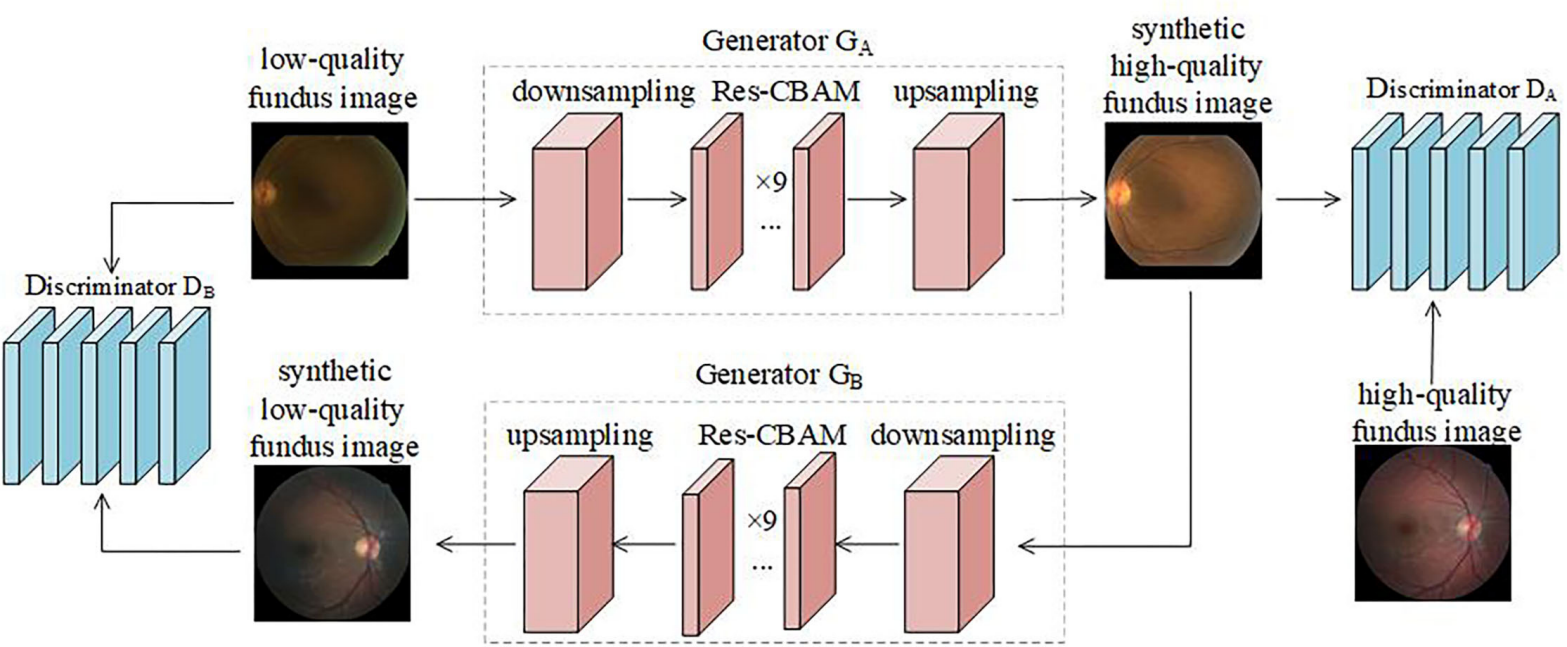
Flowchart of proposed retinal image enhancement algorithm.

Generative adversarial network is a depth model that executes a variety of image processing tasks (31). It contains a generative network that captures data distribution and a discriminative network that determines the probability that an image originates from real images. Based on GAN, CycleGAN trains the generator and discriminator alternately, essentially acting as two mirror-symmetric GANs forming a ring network. In CycleGAN, there are two generators, *G*:*A*→*B* and *F*:*B*→*A*, and two discriminators, *D*_*A*_ and *D*_*B*_. CycleGAN employs the concept of cycle consistency loss. As shown in Figure 2, the low-quality retinal image inputs *G*_*A*_ to generate high-quality images, which then inputs *G*_*B*_, and are converted back into the low-quality retinal images. This is a cyclic process of retinal images alternating from low to high quality. The reconstructed low-quality image must be the same as the original image.

**Figure 3:**
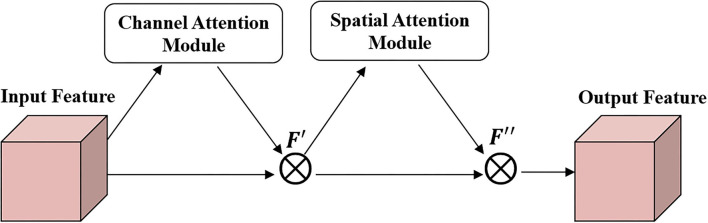
The structure of convolutional block attention modules (CBAM).

Generators, *G*_*A*_ and *G*_*B*_, are fully convolutional networks, which can be divided into a downsampling module, Res-CBAM module, and upsampling module according to the function. The downsampling module converts the information contained in the input image into a feature vector. It contains three convolutional layers, and the numbers of convolution kernels are 64, 128, and 256, respectively. The first convolution layer uses a (7,7) convolution kernel with a stride size of 1, and the remaining two layers use a (3,3) convolution kernel with a stride size of 2. The Res-CBAM module integrates the features again to extract global high-dimensional information. CBAM adopts the attention module based on the human visual attention mechanism (38), and its structure is shown in Figure 3. The proposed method stacks nine resblocks as feature sorting networks, and each residual block uses a CBAM network.

The discriminators, *D*_*A*_ and *D*_*B*_, reduce the number of discriminant network parameters by drawing on the method of PatchGANs (39) without incorporating the CBAM network. The discriminator determines the sub-graphs of different sizes intercepted from the original image and obtains the classification result according to the principle of majority voting. The discriminator consists of five convolutional layers. The numbers of convolution kernels are 64, 128, 256, 512, and 1, respectively. The stride sizes are 2, 2, 2, 1, and 1, respectively. The role of discriminators is to discriminate whether the input is a real retinal image in the dataset or a synthetic image.

### 2.3. Retinal Vessel Segmentation Network

The length, width, and curvature of retinal vessels represent important information in the diagnosis of DR, glaucoma, and other ophthalmic diseases (40). To screen for these diseases, it is necessary to segment retinal vessels from retinal images and analyze their structural characteristics. Thick and thin vessels are present in retinal images. The identification of thin vessels is a challenging task in retinal vessel segmentation. The enhanced retinal image can improve the performance of this task (41). To verify the effectiveness of this study, enhanced retinal images were applied to the classic medical image segmentation network UNet (42) to perform retinal vessel segmentation experiments. UNet adopts an encoder-decoder structure. The encoder is composed of four layers of downsampling, each of which contains two convolution layers with kernel size of 3 and a maxpooling layer. The decoder is composed of four layers of upsampling, each of which contains a convolution layer with a kernel size of 2, a concatenate layer, and two convolution layers with kernel size of 3. Skip connections are used to concatenate each layer of the downsampling with the corresponding upsampling layer. The optimizer used here is Adam with a learning rate of 0.0001. The loss function used here is the binary-cross-entropy function.

### 2.4. Loss Function

To obtain enhanced images, we train Cycle-CBAM in a manner similar to dual learning. According to cycle consistency, the image reconstructed by the generator must be consistent with the original image. We utilize the L1 norm to represent the reconstruction loss of low/high-quality retinal images in the training set. The reconstruction loss of the generator is expressed as


(1) $$\begin{array}{l} L_{cyc}\left(F_{CBAM},G_{CBAM}\right)=E_{A}\left[||F_{CBAM}\left(G_{CBAM}\left(a\right)\right)-a|{|}_{1}\right]\\ \;+E_{B}\left[||G_{CBAM}\left(F_{CBAM}\left(b\right)\right)-b|{|}_{1}\right]. \end{array}$$

The purpose of the discriminator is to determine whether the input is a real or a generated image. The discriminator loss is a binary loss, as in


(2) $$\begin{array}{l} L_{GAN}\left(F_{CBAM},D_{A},A,B\right)=E_{A}\left[logD_{A}\left(a\right)\right]\\ \;+E_{B}\left[log\left(1-D_{A}\left(F_{CBAM}\left(b\right)\right)\right)\right], \end{array}$$

(3) $$\begin{array}{l} L_{GAN}\left(G_{CBAM},D_{B},A,B\right)=E_{B}\left[logD_{B}\left(b\right)\right]\\ \;+E_{A}\left[log\left(1-D_{B}\left(F_{CBAM}\left(a\right)\right)\right)\right]. \end{array}$$

The objective loss function is expressed as


(4) $$\begin{array}{l} \;L\left(G_{CBAM},F_{CBAM},D_{A},D_{B}\right)\\ =L_{GAN}\left(F_{CBAM},D_{A},A,B\right)+L_{GAN}\left(G_{CBAM},D_{B},A,B\right)\\ +\lambda L_{cyc}\left(F_{CBAM},G_{CBAM}\right), \end{array}$$

where λ controls the weight of reconstruction and discriminator losses. Ideally, when the input image originates from the dataset, the discriminator outputs one. Otherwise, when the input is a generated retinal image, the discriminator outputs zero. The generator generates as realistic images as possible to cause misjudgment by the discriminator. Therefore, the discriminators, *D*_*A*_ and *D*_*B*_, must maximize the objective function and the generators, *G*_*CBAM*_ and *F*_*CBAM*_, must minimize it. The reconstructed retinal images must be similar to the original images, such that the reconstruction loss *L*_*cyc*_(*F*_*CBAM*_, *G*_*CBAM*_) is minimal. The performance of the network is improved through the competition game between the generators and discriminators. Our objective function is expressed as.


(5) $$\begin{array}{l} G_{CBAM},F_{CBAM}\\ =arg\underset{G_{CBAM},F_{CBAM}}{\min}\underset{D_{A},D_{B}}{\max}L\left(G_{CBAM},F_{CBAM},D_{A},D_{B}\right). \end{array}$$

### 2.5. Statistical Evaluation Metrics

The quantitative results are analyzed by statistical methods. SD measures the extent to which data values deviate from the mean. A smaller SD represents more balanced data. The SE, also known as the root mean square error, is sensitive to values with large errors. The formulas are as follows:


(6) $$\begin{array}{l} SD=\sqrt{\frac{1}{n}\underset{i}{\sum }{\left(x_{i}-\bar{x}\right)}^{2}}, \end{array}$$

(7) $$\begin{array}{l} SE=\frac{SD}{\sqrt{n}}. \end{array}$$

where $\bar{x}$ is the average, *x*_*i*_ is the data value, and the range of *i* is [0,99]. *n* is the number of the set of data. In this study, *n* is 100. The Kolmogorov–Smirnov test is a non-parametric method used to test the distribution of data. The unpaired Student's t-test is performed to test whether the difference between the two samples is significant. If the *p*-value is significantly larger than 0.05, this means that the two samples are not considerably different.

## 3. Results

This section presents the comparison experiment of qualitative and quantitative analysis of the proposed retinal image enhancement method with CLAHE (11), Fusion-based (15), MSRCP (22), LIME (16), Cycle-GAN (31) methods, and their application in the retinal vessel segmentation network. The CLAHE used in the experiments is its specialization (11). The specialized CLAHE implements CLAHE only in the G channel rather than the whole image, based on the unique property of the retinal image that the G channel has important information. Then, the enhanced G channel is merged with the R channel and B channel to obtain the enhanced retinal image. All experiments in this study are based on the Keras framework. The computer hardware configuration is an Intel Core i7-7700k CPU, 16GB RAM, and an NVIDIA RTX 2080Ti 11GB GPU. The computer software environment is Tensorflow-gpu 1.11.0, Keras 2.2.4, CUDA 9.0, Cudnn 7.3.0, Python 3.6.4, and Opencv-Contrib-Python 3.4.2.16. The Adam optimizer is used in the network training process; the learning rate is 0.0002, and the number of training rounds is 200.

### 3.1. Qualitative Analysis Results

**Figure 4:**
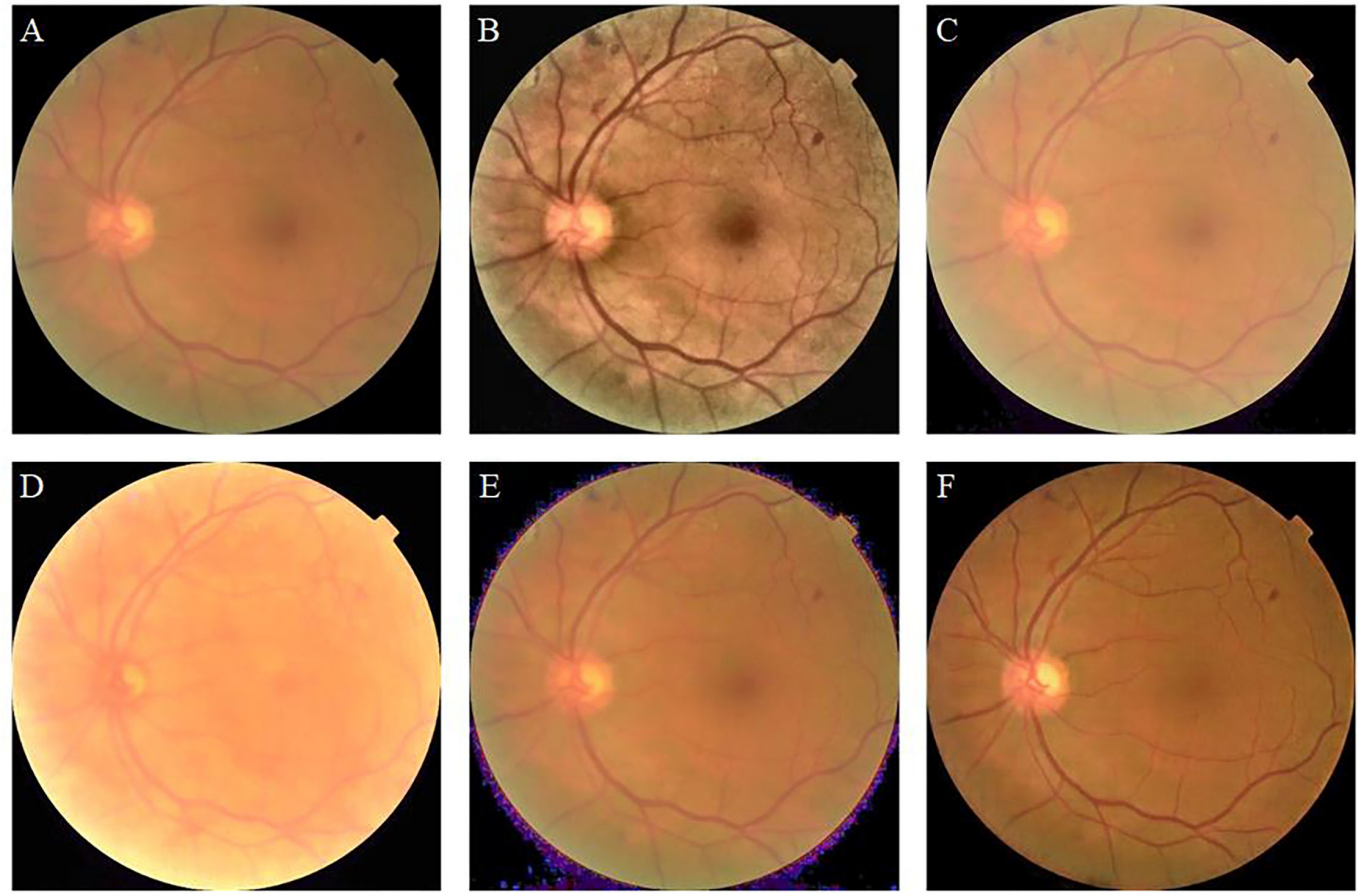
Blurry retinal image and enhancement results of different methods. **(A)**: Original image. **(B)**: contrast limited adaptive histogram equalization (CLAHE). **(C)**: Fusion-based. **(D)**: MSRCP. **(E)**: low-light image enhancement (LIME). **(F)**: Ours.

In the retinal image with a resolution of 512 × 512, the width of the thickest retinal vessel is only 11 pixels, and the blurred retinal image does not clearly show the vessels (Figure 4A). The image enhanced by CLAHE shows thickened retinal vessels, which indicate the clinical manifestation of hypertensive retinopathy. The image appears green at the edges and near the blood vessels (Figure 4B). The enhanced image using the fusion-based method is not sufficiently clear and seems to be shrouded in fog (Figure 4C). The MSRCP algorithm over-enhanced the image, resulting in exposure at the bottom edge. The blood vessels are bright red, and the optic disc area is blurred (Figure 4D). The LIME method does not effectively improve the clarity and produces noise at the boundary between the foreground and the background (Figure 4E). Our algorithm improves the visual resolution of retinal images and maintains the original structure of the blood vessels (Figure 4F). The clear boundary between the optic cup and optic disc is convenient for pathological analysis by ophthalmologists and CAD systems.

**Figure 5:**
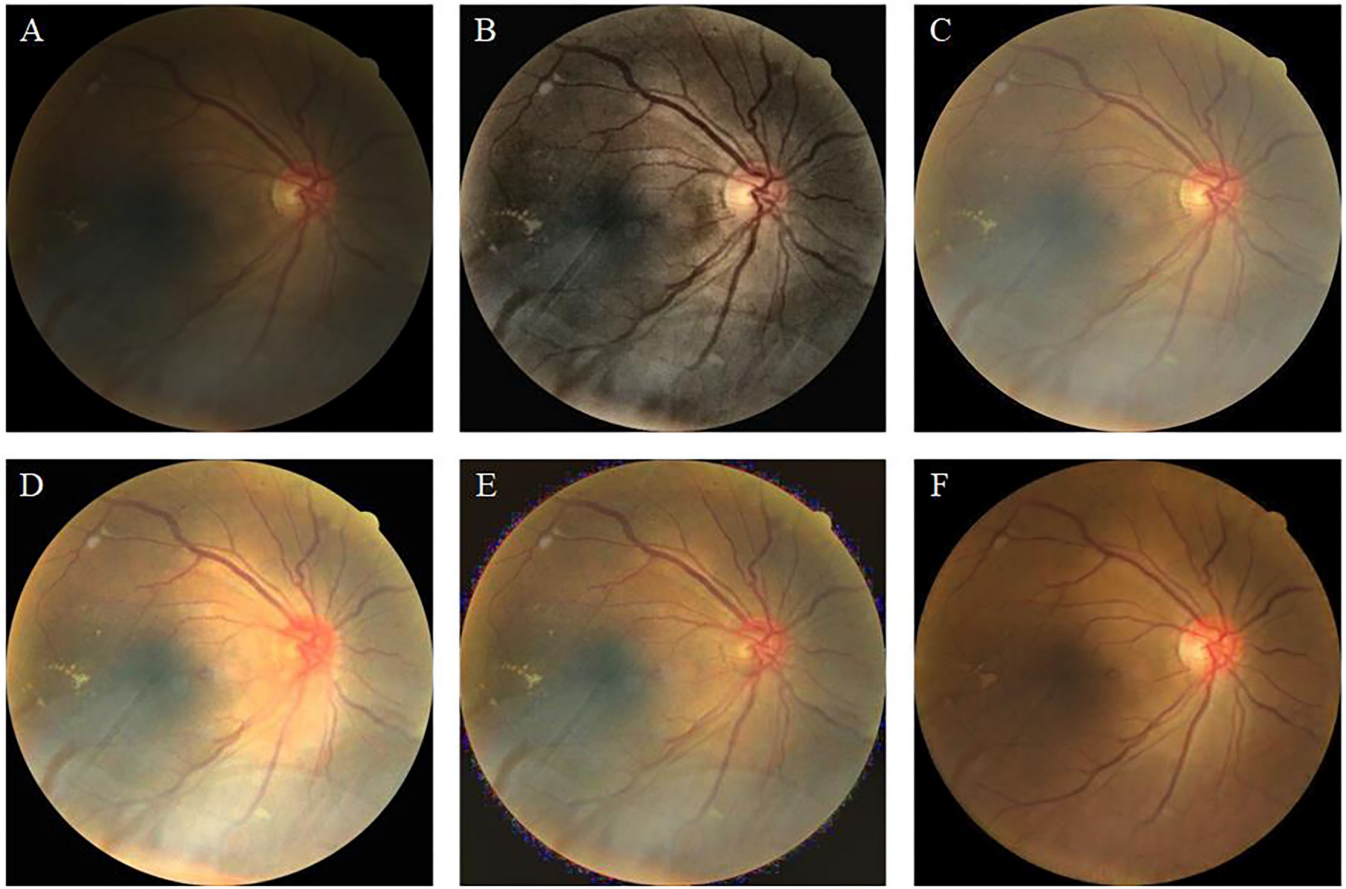
Low-illumination retinal image and enhancement results of different methods. **(A)**: Original image. **(B)**: CLAHE. **(C)**: Fusion-based. **(D)**: MSRCP. **(E)**: LIME. **(F)**: Ours.

Figure 5 shows the enhancement results of different algorithms on low-illumination retinal images. Figure 5B appears green overall, which is inconsistent with the natural condition of the retina. The overall brightness of Figures 5C–E is improved; however, eyelash artifacts obscure the lower half of the image. In addition, exposure occurs around the optic disc in Figure 5D, and the left edge of the optic disc cannot be observed. In contrast, our algorithm (Figure 5F) significantly improves image brightness and contrast. The image enhanced by our algorithm eliminates eyelash artifacts and enables the clear display of blood vessels, the optic disc, and macula.

**Figure 6:**
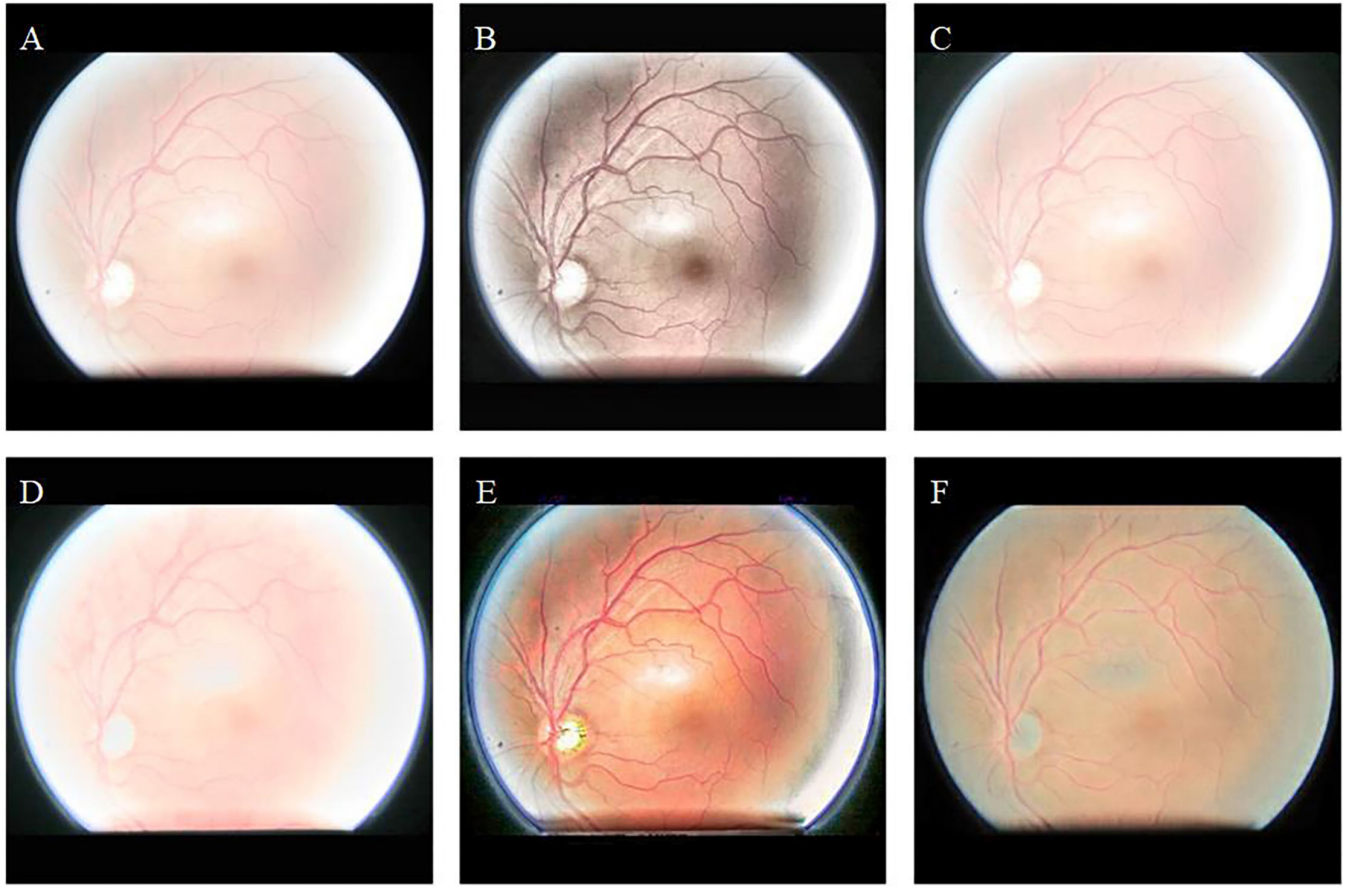
High-illumination retinal image and enhancement results of different methods. **(A)**: Original image. **(B)**: CLAHE. **(C)**: Fusion-based. **(D)**: MSRCP. **(E)**: LIME. **(F)**: Ours.

Figure 6 shows the high-illumination retinal image and the enhancement results achieved by different methods. The image enhanced by CLAHE shows pink around the image and the macula, which is the darker area of the original image (Figure 6B). The brightness and contrast of the images enhanced by fusion-based (Figure 6C) and MSRCP (Figure 6D) methods have not been improved, and there seems to be no enhancement compared with the original image. This indicates that these two methods cannot enhance high-light retinal images. The LIME method excessively restores the color of the image, and the high saturation masks the color information of the original image (Figure 6E). Our method (Figure 6F) restores the blood vessels in red while retaining the color saturation of an original image, which is closer to the real situation of the retina.

**Figure 7:**
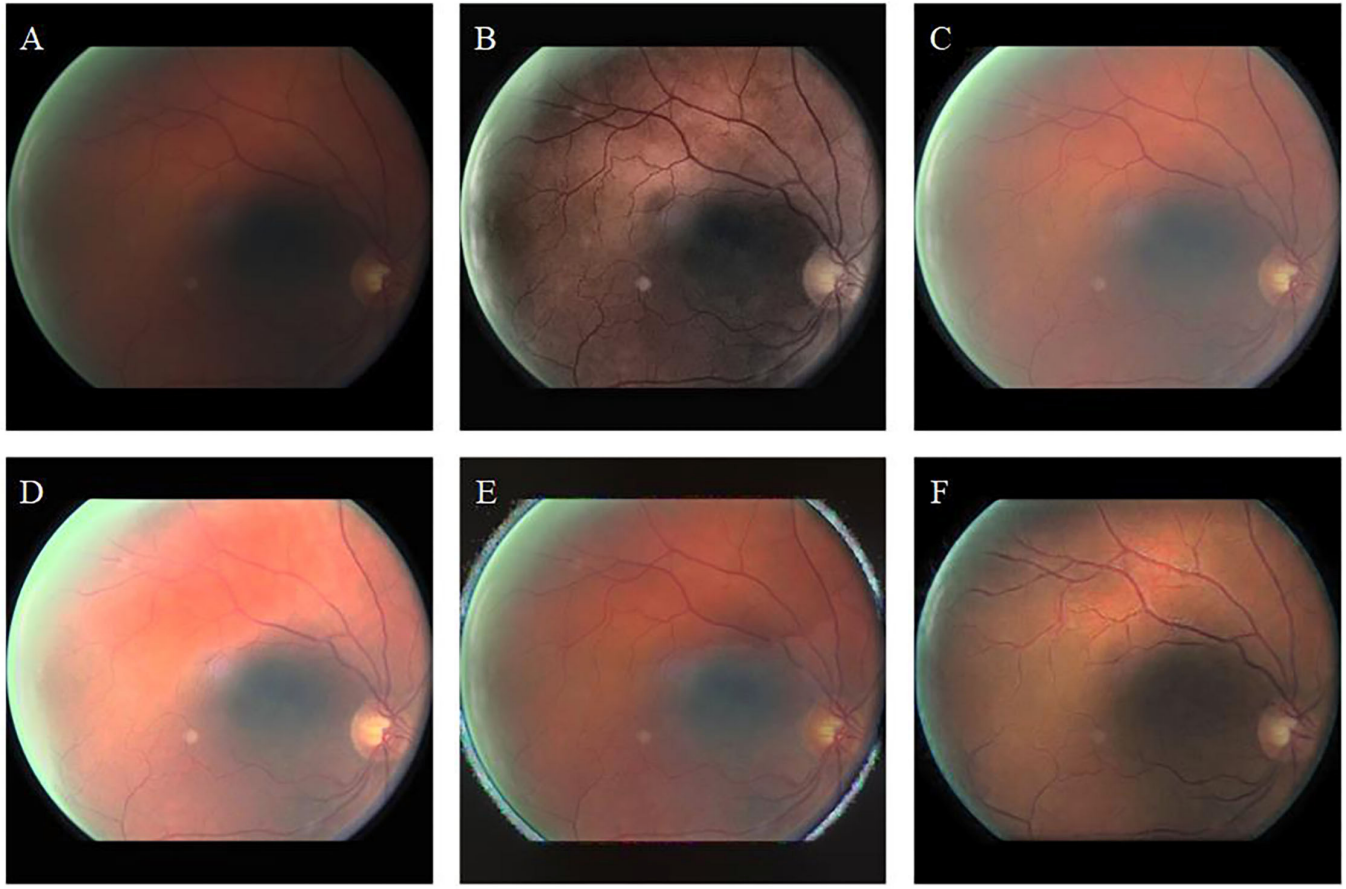
Uneven-illumination retinal image and enhancement by different methods. **(A)**: Original image. **(B)**: CLAHE. **(C)**: Fusion-based. **(D)**: MSRCP. **(E)**: LIME. **(F)**: Ours.

Figure 7 shows the uneven-illumination retinal image and enhancement results obtained by different methods. The CLAHE method improves the contrast of the original image; however, the visibility in low light remains low (Figure 7B). The fusion-based method improves the overall brightness of the image; however, the visual resolution of the enhanced image is poor (Figure 7C). In the MSRCP-enhanced image, blood vessels in low-light areas appear black, and blood vessels in high-light areas appear red (Figure 7D). The color of retinal blood vessels changes which is not consistent with the real retina. The LIME method generates noise at the junction of the foreground and background (Figure 7E). Our algorithm (Figure 7F) improves the overall brightness and contrast of the image and maximizes the clarity of thin blood vessels.

**Figure 8:**
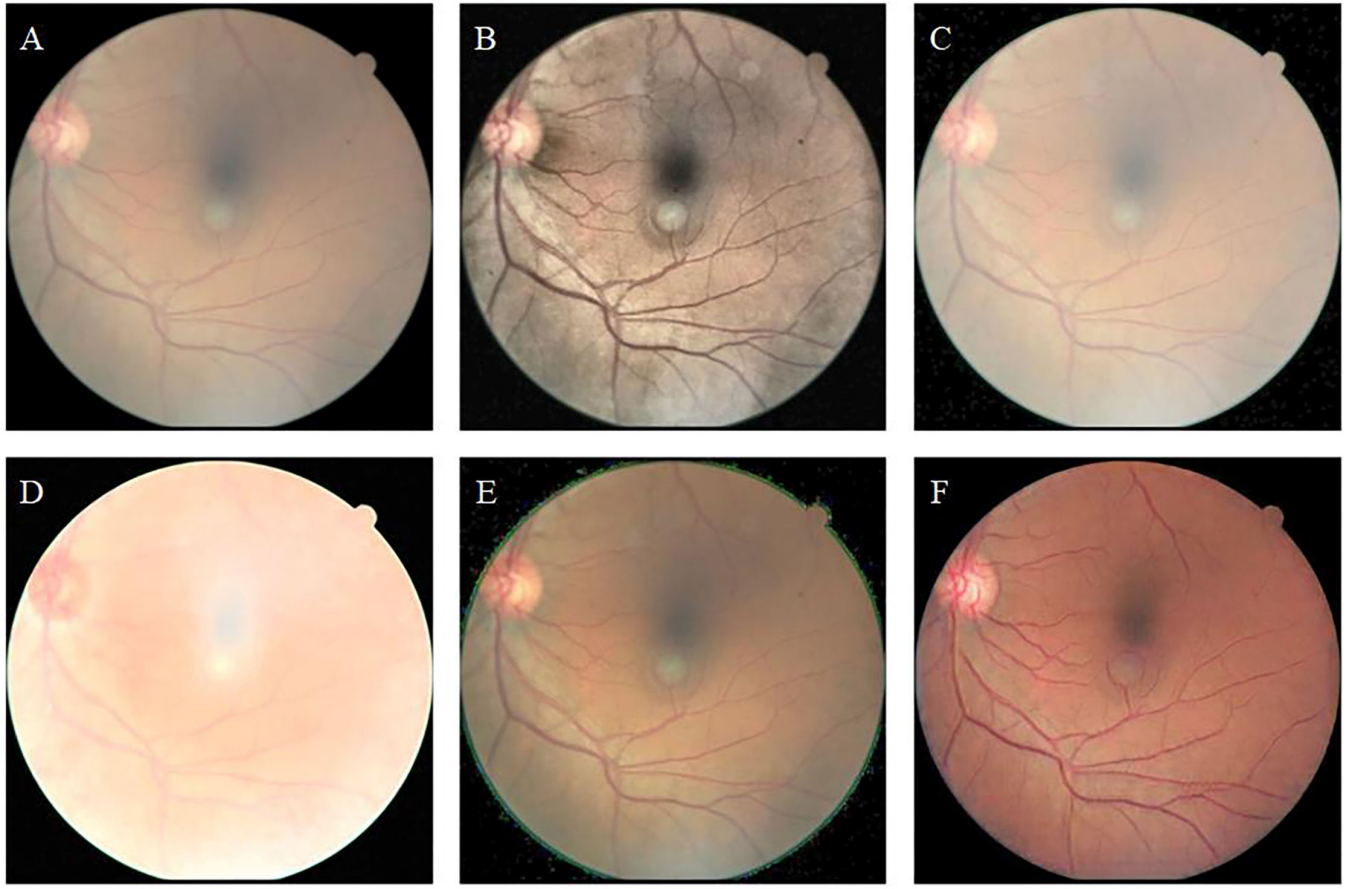
Color-distorted retinal image and enhancement results of different methods. **(A)**: Original image. **(B)**: CLAHE. **(C)**: Fusion-based. **(D)**: MSRCP. **(E)**: LIME. **(F)**: Ours.

Figure 8 shows the color-distorted retinal image and the enhancement results obtained by different methods. The original image (Figure 8A) has a whitish tone, which does not match the red of the real retina. (Figures 8B–E) adjusts the brightness or contrast; however, none of them improve the color tone of the original image. Among them, MSRCP (Figure 8D) causes overexposure due to excessive brightness enhancement. The images enhanced by our method (Figure 8F) show red tones, which are closer to the real retinal situation. Our algorithm restores color information with high color saturation, where other algorithms fail.

### 3.2. Comparison Results Between CycleGAN and Cycle-CBAM

**Figure 9:**
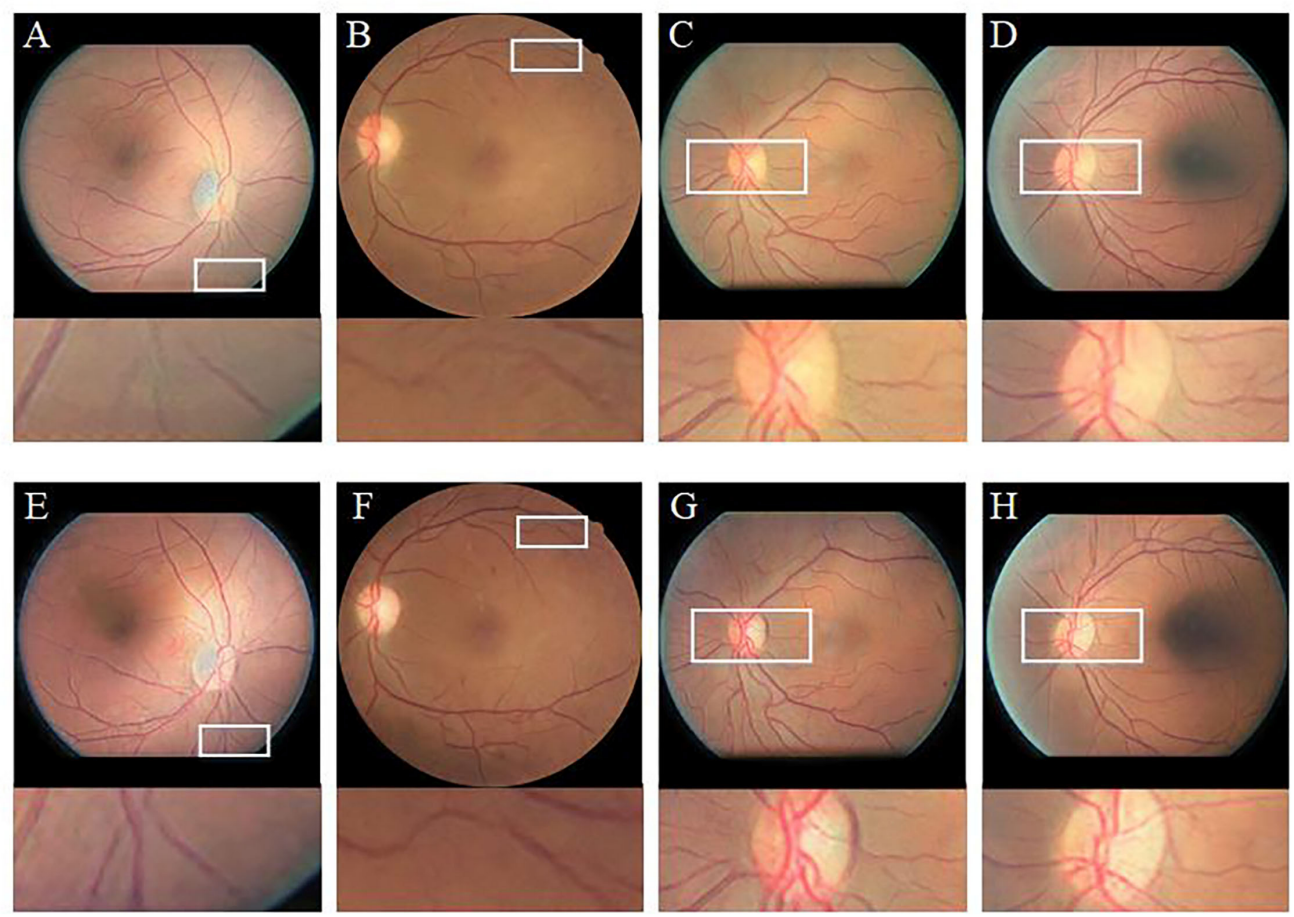
Visual comparison of enhancement results of CycleGAN and Cycle-CBAM on retinal images. **(A–D)**: Enhanced by CycleGAN. **(E–H)**: Enhanced by Cycle-CBAM.

To verify the effectiveness of the CBAM module, we compare the enhancement results of CycleGAN (without CBAM) and Cycle-CBAM (our method) (Figure 9). The first row shows the CycleGAN-enhanced images with zoomed-in views of selected regions (white rectangles). The second row depicts the corresponding images enhanced by Cycle-CBAM. The selected areas mark the ends of blood vessels (the first two columns) or the optic disc (the last two columns). The CycleGAN-enhanced blood vessels (Figures 9A,B) are not distinguishable and fractured, making it difficult for the ophthalmologist to observe the morphology of the endings. At the same position in the same image, the blood vessels enhanced by Cycle-CBAM (Figures 9E,F) are coherent and have clear textures. The optic disc has dense blood vessels and is where the main blood vessels are in confluence. In Figures 9C,D, blood vessels are disconnected at the edge of the optic disc, which does not conform to the continuity of the blood vessels. The intersection of vessels is blurred and cannot be diagnosed. In Figures 9G,H, the edges of the optic disc are easily distinguishable, and the blood vessels are coherent and clear. Cycle-CBAM enhanced images have reddish colors and high saturation.

### 3.3. Quantitative Analysis Results

**Figure 10:**
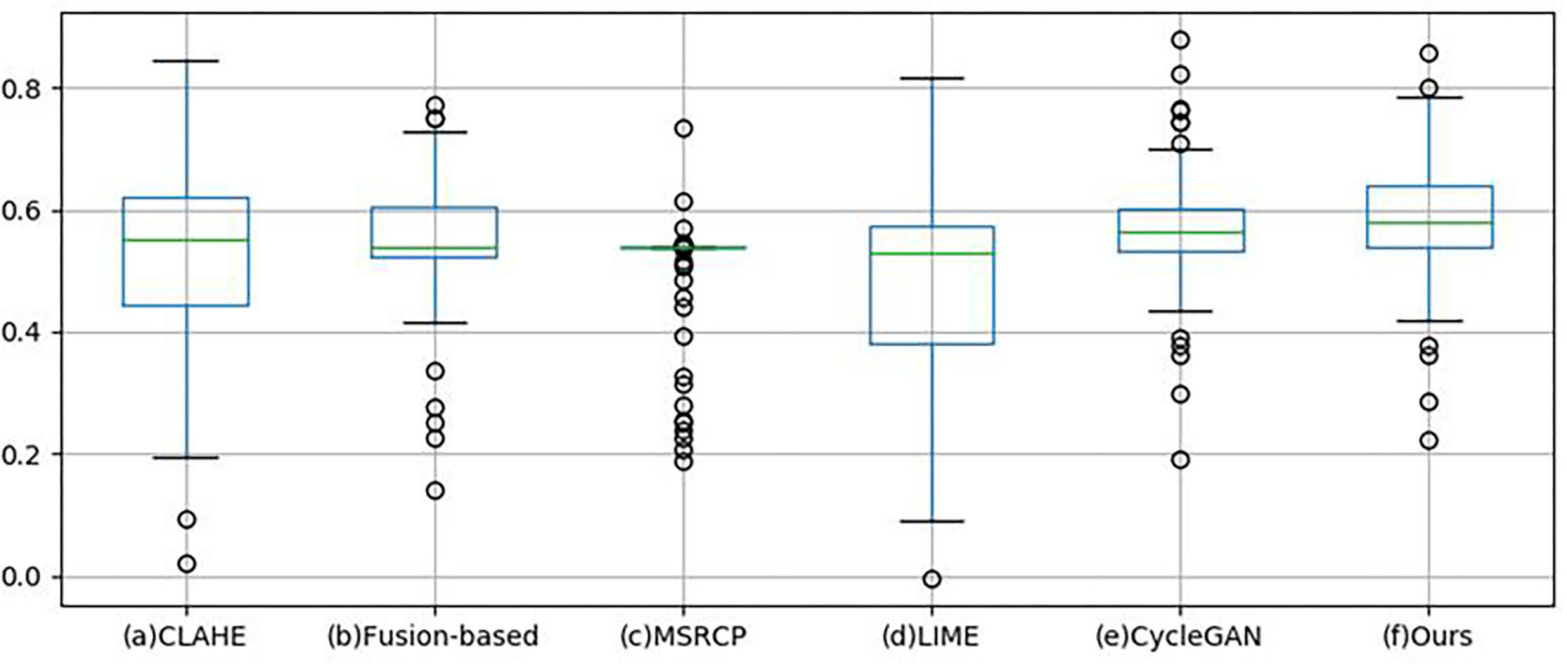
Box-plot of Blind/Referenceless image spatial quality evaluator (BRISQUE).

**Figure 11:**
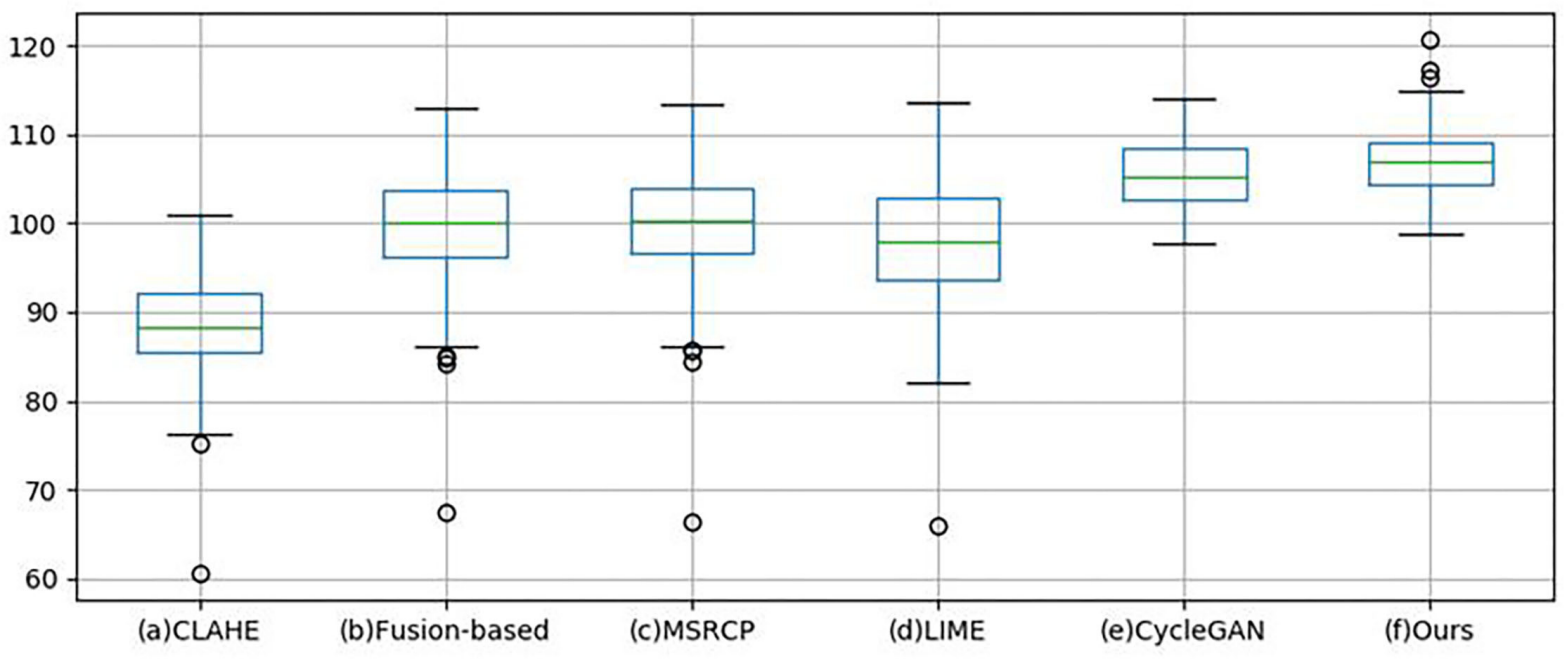
Box-plot of HUE.

Quantitative analysis is carried out using non-reference and full-reference image quality assessment. For non-reference metrics, Blind/Referenceless image spatial quality evaluator (BRISQUE) (43) and HUE are used to evaluate the enhanced images of 100 retinal images in the test set by different methods. Brisque is an image quality assessment metric, and HUE adopts the HSV color space. Table 2 shows the quantitative evaluation results of non-reference metrics. BRISQUE and HUE scores are obtained for each image, and the average value is taken as the final score of 100 images. Subsequently, statistical significance tests were performed on the BRISQUE and HUE scores. Our approach achieved the highest BRISQUE score, which indicates a superior image quality. The image quality enhanced by our method is balanced with an SD of 0.1006 and an SE of 0.0071. In the box-plot (Figure 10), the distribution of image scores enhanced by our method is concentrated, and the median (green line) is higher than that of other methods, which is consistent with the results presented in Table 2. The MSRCP algorithm has the most concentrated scores; however, it has a large number of outliers (the circles in Figure 10). In the unpaired Student's t-test, a *p*-value larger than 0.05 indicates that there is no significant difference between enhanced images and original images. The *p*-values of the CLAHE, Fusion-based, CycleGAN, and Cycle-CBAM are 0.8561, 0.1703, 0.8198, and 0.3505, respectively, which do not differ significantly from the original images. In contrast to traditional algorithms, the proposed deep learning algorithm can retain pixel-level details. Cycle-CBAM also achieved the highest HUE score, which means it has the best color information. In Figure 11, our upper and lower quartiles (the upper and lower edges of the box) are higher than those in other algorithms, and the data distribution is concentrated. For the Kolmogorov–Smirnov test, the hue scores follow a normal distribution. The SD and SE of Cycle-CBAM are 3.9964 and 0.2826, respectively, both of which are lower than those of traditional algorithms. In the unpaired Student's t-test, the *p*-values of CLAHE and Cycle-CBAM are close to zero, indicating that the hue of the enhanced image significantly differs from that of the original.

**Table 2:** Evaluation results of non-reference metrics.

**Metrics**		**CLAHE**	**Fusion-based**	**MSRCP**	**LIME**	**CycleGAN**	**Cycle-CBAM**
BRISQUE	score	0.5699	0.5549	0.5120	0.4772	0.5696	0.5836
	SD	0.1095	0.1065	0.0879	0.1624	0.0946	0.1006
	SE	0.0077	0.0075	0.0062	0.0115	0.0067	0.0071
	*p*	0.8561	0.1703	0.0000	0.0000	0.8198	0.3505
HUE	score	86.2863	99.4257	99.9469	98.0184	105.5443	106.9730
	SD	7.2309	7.0586	7.2729	8.0034	3.5790	3.9964
	SE	0.5113	0.4991	0.5143	0.5659	0.2531	0.2826
	*p*	0.0000	0.5121	0.8868	0.0573	0.0000	0.0000

Peak signal to noise ratio (PSNR) (44) and structural similarity (SSIM) (45) are commonly used full-reference metrics for evaluating images. In our study, these two metrics are used to evaluate the cataract images of the proprietary dataset (Table 3). The high-quality image taken in the same position after the operation is taken as the ground truth. The larger the PSNR, the smaller the image distortion, whereas a larger SSIM indicates a higher image similarity. Our method obtains optimal values in both the metrics, indicating that the enhanced image exhibits the least distortion and best preservation of the structure of the original image. It takes an average of 35 ms to enhance each image, which is faster than traditional algorithms.

**Table 3:** Evaluation results of full-reference metrics.

**Metrics**	**CLAHE**	**Fusion-based**	**MSRCP**	**LIME**	**CycleGAN**	**Cycle-CBAM**
PSNR	19.0088	17.5748	11.5473	21.2084	22.4953	24.7386
SSIM	0.5920	0.7693	0.6452	0.7762	0.7643	0.8103
Time (ms)	40	4820	1010	1302	35	35

The enhancement of retinal images serves to assist doctors in pathological analysis. Therefore, the quality of the enhanced images was evaluated by three ophthalmologists. The ophthalmologists determined the quality of the enhanced images as follows: the main structures and lesion areas of the original images were not lost, added, or changed, and were clearly visible. Each of the 100 images in the test set corresponds to six enhancement results. Each ophthalmologist independently voted for the best enhanced image. Each doctor held 100 votes; hence, the three doctors held a total of 300 votes. The votes of the three ophthalmologists were accumulated to obtain the final votes for the six methods (Table 4). The result shows that our method obtained 61% of the votes, which is significantly higher than the other five methods.

**Table 4:** Subjective evaluation by ophthalmologists.

**Method**	**CLAHE**	**Fusion-based**	**MSRCP**	**LIME**	**CycleGAN**	**Cycle-CBAM**
vote	0	20	14	19	64	183
proportion	0	6.67%	4.67%	6.33%	21.33%	61%

We also applied these images to the deep learning model Multiple Color-Space Fusion Network (MCF-Net) (46) for quality assessment. The model integrates RGB, HSV, and Lab color spaces to categorize the quality of retinal images into good, usable, and reject. Retinal images with clear features of retinopathy are classified as good. Images that are not of good quality but have clear primary structures, such as blood vessels, macula, and optic disc, are sorted as usable. Images that are so poor in quality that cannot be used by ophthalmologists for diagnosis are classified as reject. Table 5 shows the results of quality assessment of retinal images enhanced by different methods. The good, usable, and reject categories of the original images are 0%, 34%, and 66%, respectively. Compared with the other five image enhancement methods, Cycle-CBAM method is the best, with the largest number of good images and the least number of reject images. For the images enhanced by traditional methods, there are less than 10% good images, while the reject grade still accounts for more than half. However, of the images enhanced by the proposed method, 57% were classified as good and the number of reject images reduced from 66 to 12%.

**Table 5:** Quality assessment by Multiple Color-Space Fusion Network (MCF-Net).

**Method**	**Original**	**CLAHE**	**Fusion-based**	**MSRCP**	**LIME**	**CycleGAN**	**Cycle-CBAM**
Good (%)	0	3	7	6	7	45	57
Usable (%)	34	44	41	20	42	38	31
Reject (%)	66	53	52	74	51	17	12

### 3.4. Results of Retinal Vessel Segmentation Application

**Figure 12:**
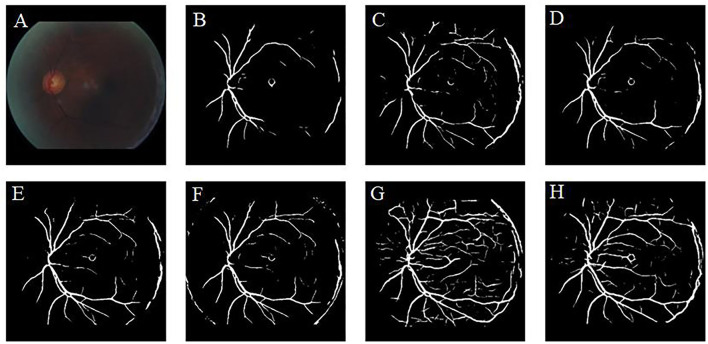
Results of retinal vessel segmentation in the low-illumination and enhanced images by different methods. **(A)**: Low-illumination image. **(B)**: Segmentation result of the low-illumination image. **(C–H)**: Segmentation results of images enhanced by different methods. [**(C)**: CLAHE. **(D)**: Fusion-based. **(E)**: MSRCP. **(F)**: LIME. **(G)**: CycleGAN. **(H)**: Ours].

**Figure 13:**
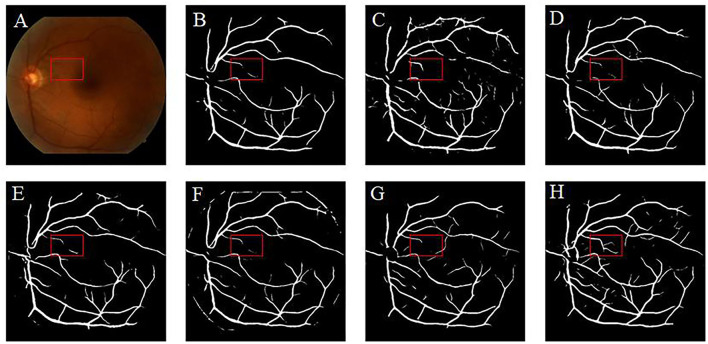
Results of retinal vessel segmentation in the blurred and enhanced images by different methods. **(A)**: Blurred image. **(B)**: Segmentation result of the blurred image. **(C–H)**: Segmentation results of images enhanced by different methods. [**(C)**: CLAHE. **(D)**: Fusion-based. **(E)**: MSRCP. **(F)**: LIME. **(G)**: CycleGAN. **(H)**: Ours].

Retinal vessel segmentation is crucial for the screening of eye diseases. To verify the effectiveness of image enhancement, we applied the enhanced retinal images to the retinal vessel segmentation task. Figure 12A shows the low-illumination retinal image, where only a few large vessels were recognized with short vessel lines (Figure 12B). Images enhanced by CycleGAN (Figure 12G) and Cycle-CBAM (Figure 12H) can be segmented into complete blood vessels. In Figure 12G, there are some messy thin blood vessels, which are not continuous. This may be caused by the misidentification of noise in the enhanced image. Figure 12H can accurately segment the thick and thin blood vessels. The veins are coherent and can reveal the true structure of blood vessels. Figure 13 demonstrates the results of retinal vessel segmentation of blurred image and images enhanced by different methods. Figure 13B shows only the main blood vessels, which can also be easily identified by the naked eye in Figure 13A. The results of the enhanced images (Figures 13C–G) show more blood vessels, but the segmentation of small vessels is insufficient. By comparing the red boxes at the same position in each subgraph, only the proposed method (Figure 13H) can clearly display the complete vessel structure and show the small vessels in the red box, which indicates the successful application of the proposed method in retinal vessel segmentation and the effectiveness of our enhancement method.

Table 6 lists the accuracy of vessel segmentation when images enhanced by different methods were applied. High-quality represents the high quality of the original images of the datasets, and low-quality represents the low-quality of the retinal images generated by Cycle-CBAM. We trained the network independently using the DRIVE, STARE, and CHASEDB1 datasets. Compared with the other enhancement methods, the proposed algorithm achieved the highest accuracy on all the datasets, and the accuracy values were 0.9612, 0.9649, and 0.9669, respectively. The accuracy was even higher on the STARE and CHADEDB1 datasets compared to the original high-quality images (the ones in bold denote the best values).

**Table 6:** Accuracy of retinal vessel segmentation on public datasets.

**Database**	**DRIVE**	**STARE**	**CHASEDB1**
High-quality	**0.9624**	0.9560	0.9631
Low-quality	0.9327	0.9419	0.9501
CLAHE	0.9516	0.9455	0.9578
Fusion-based	0.9580	0.9521	0.9618
MSRCP	0.9431	0.9500	0.9573
LIME	0.9568	0.9489	0.9591
CycleGAN	0.9455	0.9476	0.9575
Cycle-CBAM	0.9612	**0.9649**	**0.9669**

*The bold values denote the best values*.

## 4. Discussion

Color retinal images are the most commonly used imaging data for screening and diagnosing ophthalmic diseases, and they are usually captured using fundus cameras. Because of factors such as exposure discomfort, equipment parameter setting errors, improper operation, and varying medical staff experience during the image acquisition process, there are many low-quality retinal images in the current retinal image database. Retinal image enhancement can improve the quality of the retinal image database and be used to train high-quality diagnostic models. It can also improve the quality of retinal images collected in ophthalmology clinics for artificial intelligence analysis and clinical diagnosis.

Our literature review indicates that learning-based methods have been barely explored for the enhancement of retinal images. In this study, a deep learning method was used to enhance various types of low-quality retinal images to improve their quality. Similar to the public dataset, the dataset used in this study contains retinal images taken before and after cataract surgery collected from the clinical work of the research team. This study found that CLAHE improves the overall contrast of the image; however, it thickens the blood vessels. Changes in the original shape of blood vessels may lead to undesirable results in the CAD system. The CLAHE method showed significant differences in the HUE metric from the original image, but visual analysis showed that these differences are caused by the excessive increase of gray information. These images did not receive the votes of fundus doctors in the voting experiment. The *p*-value of the fusion-based method in the BRISQUE is larger than 0.05 (*p* = 0.1703), which is significantly different from the original image. However, fusion-based and MSRCP methods improve the image quality by adjusting the illumination, whereas they cannot enhance blurred and high-illumination retinal images. LIME has good enhancement results for uneven-illumination images; however, it produces noise at the junction of foreground and background, which will interfere with the CAD system diagnosis of diseases. Traditional methods cannot restore the color of the images. Our method even succeeds in enhancing the color distortion of retinal images. Figure 9 shows that the introduction of the CBAM module enables the network to have stronger feature extraction capabilities and improves the ability to enhance details, such as tiny blood vessels and optic discs. The extraction of color is also significantly improved, which is crucial for color retinal images. The images enhanced by the proposed method can be segmented into complete blood vessels, exhibiting good performance in the task of segmentation (Figures 12, 13).

## 5. Conclusion

This study adopts cycle-constraint adversarial network CycleGAN to realize retinal image enhancement. To improve feature extraction and detail representation, CBAM is embedded in the main architecture. This method breaks through the limitation of current image enhancement algorithms, which only succeed at enhancing a single type of low-quality retinal image. Our deep learning method overcomes the shortcomings of traditional methods, such as color distortion and complex calculations. The proposed method does not require paired images and addresses the problem of finding a large number of paired low/high-quality retinal images. In future studies, we aim to collect more types of retinal images in clinics and attempt to repair defects, such as bright spots and eyelash artifacts. We plan to integrate the retinal image enhancement network with the classification network to build an end-to-end fundus disease diagnosis system.

## Data Availability Statement

The original contributions presented in the study are included in the article/supplementary material, further inquiries can be directed to the corresponding author/s.

## Author Contributions

CW and XZ: acquired, analyzed, explained the data, and drafted the manuscript. QY and JS: designed the study. JS, SZ, QJ, and WY: acquired the clinical information and revised the manuscript. All authors contributed to the article and approved the submitted version.

## Funding

Chinese Postdoctoral Science Foundation (2019M661832); Jiangsu Planned Projects for Postdoctoral Research Funds (2019K226); Jiangsu Province Advantageous Subject Construction Project; the Nanjing Enterprise Expert Team Project.

## Conflict of Interest

The authors declare that the research was conducted in the absence of any commercial or financial relationships that could be construed as a potential conflict of interest.

## Publisher's Note

All claims expressed in this article are solely those of the authors and do not necessarily represent those of their affiliated organizations, or those of the publisher, the editors and the reviewers. Any product that may be evaluated in this article, or claim that may be made by its manufacturer, is not guaranteed or endorsed by the publisher.
